# The Association in Myopic Tractional Maculopathy With Myopic Atrophy Maculopathy

**DOI:** 10.3389/fmed.2021.679192

**Published:** 2021-08-20

**Authors:** Jiaxin Tian, Yue Qi, Caixia Lin, Kai Cao, Ningli Wang

**Affiliations:** Beijing Tongren Eye Center, Beijing Institute of Ophthalmology, Beijing Ophthalmology and Visual Sciences Key Laboratory, Beijing Tongren Hospital, Capital Medical University, Beijing, China

**Keywords:** association, myopic tractional maculopathy, myopic atrophy maculopathy, characteristics, fundus images, optical coherence tomography

## Abstract

**Purpose:** To investigate the relationship between myopic tractional maculopathy (MTM) and myopic atrophy maculopathy (MAM).

**Method:** Two hundred and six eyes with definitive myopic retinoschisis were assessed in the retrospective observational case series study and the atrophic and tractional features were further evaluated. Atrophic changes were analyzed according to the atrophic component in the ATN classification and the occurrence of gamma zones and delta zones. Tractional changes were evaluated based on different retinoschisis layers, the location and range of outer retinoschisis, retinal detachment, inner lamellar macular hole (ILMH), outer lamellar MH (OLMH), full-thickness MH (FTMH), and paravascular abnormalities.

**Results:** Of all the eyes, 29.6, 42.7, 19.4, and 8.3% presented MAM grades with A1, A2, A3, and A4, respectively. The three layers of retinoschisis and the entire macular retinoschisis had the highest incidences in A2 (38.6%; 54.5%). The numbers of retinoschisis layers and the grades of outer retinoschisis had a weak negative correlation with MAM (*r* = −0.138, *P* = 0.048; *r* = −0.139, *P* = 0.047). All the eyes had gamma zones, and 82.52% of eyes also had delta zones. The incidence of retinal detachment and OLMH reached the peak in A2 and then decreased gradually. With MAM aggravation, the prevalence of ILMH decreased. Eyes with A1 and A2 were more likely to have OLMH, and those with A3 and A4 were more likely to have FTMH (*P* = 0.028; OR, 3.423; 95% CI, 1.144–10.236; *P* = 0.004; OR, 7.752; 95% CI, 1.951–30.803). With the MAM grades growing, the types of paravascular abnormalities increased (*r* = 0.165, *P* = 0.018).

**Conclusion:** Diffuse chorioretinal atrophy was the dominant MAM grade in eyes with MTM. In the study, 72.3% of eyes with MTM presented with diffuse chorioretinal atrophy and a tessellated fundus. Over 80% of eyes with MTM had both gamma zones and delta zones. Diffuse chorioretinal atrophy might be a complicated stage for MTM with the highest rate of three layers of retinoschisis, the entire macular retinoschisis, RD, and OLMH. Atrophic progression might involve the development of MH. When MTM combines with well-defined atrophy, the occurrence of FTMH should be noted.

## Introduction

As we all know, in combination with environmental changes, lifestyle, and many other factors, the prevalence of myopia is showing significant and sustained growth worldwide (1, 2). Following that, pathologic myopia and myopic macular degeneration are also increasing year by year and becoming a dominant cause of blindness (3). As an acquainted complication in highly myopic eyes, investigators first described myopic retinoschisis (MR) in 1938 (4). It was also regarded as retinal detachment (RD) without a hole in a myopic patient with posterior staphyloma in 1958 (5). In 1999, Takano and Kishi disclosed foveal retinoschisis by optical coherence tomography (OCT) in pathologic myopia patients (6). Later, Panozzo and Mercanti proposed myopic traction maculopathy (MTM), which included different stages of tractional maculopathy, like MR, RD, and macular hole (MH) (7).

Currently, investigators tend to divide myopic maculopathy into three components, myopic atrophy maculopathy (MAM), MTM, and myopic neovascular maculopathy. This classification helps us gain a comprehensive understanding of the disease and clinical management (8). As an essential part of pathologic myopia, the MAM grade reveals a general fundus condition and involves different types of myopic retinopathy. In the previous studies, people believed that MTM always occurred in the advanced stage of pathologic myopia with severe atrophic changes (9). In the study by Chen et al. there was no direct relationship between MAM and MTM based on the ATN system (10). At present, the characteristics of atrophy in MTM are not explicit. Besides, from the integrity of myopic retinopathy, both kinds of pathological changes could exist in the meanwhile. We speculate that there could be some interaction between MAM and MTM. Therefore, we made a detailed analysis of atrophic and tractional changes in eyes with definite MR to evaluate the relationship between MAM and MTM.

## Materials and Methods

### Patients Enrolled and Ocular Examination

In this retrospective observational case series study, we reviewed highly myopic patients with an axial length >26.5 mm or a refractive error < -6 diopters in Beijing Tongren Hospital from October 2018 to October 2020. Eyes with definite MTM (MR and the complication stage of MTM, including RD and MH) were enrolled in the study. The exclusion criteria were as follows: vitreoretinal surgery history; combined with other ocular diseases involving fundus changes and retinoschisis, including age-related macular degeneration, diabetic retinopathy, retinal vein occlusion, glaucoma, uveitis; subjects without available fundus or OCT images, like opacities of refractive media, and images with poor quality. The study adhered to the tenets of the Declaration of Helsinki and was approved by the Ethics Committee of Beijing Tongren Hospital.

All patients underwent regular ophthalmologic examinations, including slit-lamp biomicroscopy, fundoscopy, refraction with an assessment of best-corrected visual acuity (BCVA), A-mode ultrasonography for measurement of axial length (AXL), intraocular pressure, and fundus photography (fundus camera TRC-50; Topcon, Tokyo, Japan). In addition, two kinds of spectral-domain OCT images covering a macular area were obtained. One (RTVue-XR Avanti Optovue, Inc., Fremont, CA, United States) was performed with 10 mm scans along 18 meridians centered on the fovea. The other one (Spectralis, Heidelberg Engineering Co., Heidelberg, Germany) was measured with horizontal and vertical scans in the 11.5 × 11.5 mm rectangle area. Both measuring modes could accurately reflect fundus changes.

### Images Analysis

We evaluated MAM in fundus images based on the ATN system's atrophic component as follows: A0: no myopic retinal degenerative lesion; A1: tessellated fundus; A2: diffuse chorioretinal atrophy; A3: patchy chorioretinal atrophy; A4: macular atrophy (8). For parapapillary atrophy, we recorded the presence of gamma zones and delta zones according to the previous method (11).

In OCT images, we defined three layers of retinoschisis according to the previous study: inner retinoschisis (schisis between ILM and ganglion cell layer), middle retinoschisis (schisis in inner plexiform layer and/or inner nuclear layer), and outer retinoschisis (schisis in the outer plexiform layer) (12). We followed the classification proposed by Shimada et al. to assess the range and location of outer retinoschisis: S0, no macular retinoschisis; S1, extra-foveal; S2, fovea-only; S3, foveal but not entire macular area; and S4, entire macular area. If there was no outer retinoschisis, but inner retinoschisis and/or middle retinoschisis existed, we classified it into S0 (13). Besides, we recorded the occurrence of complications of MR in MTM, including RD, MH, and further divided MH into inner lamellar MH (ILMH), outer lamellar MH (OLMH), and full-thickness MH (FTMH). Paravascular abnormalities (PVAs), including paravascular microfolds, paravascular retinal cysts, and paravascular lamellar holes, were also identified (14–16).

All the images were analyzed by two ophthalmologists (J.X.T. and C.X.L.) independently. For results with disagreement, the more experienced retinal specialist (Y.Q.) was consulted to make a final decision.

### Statistical Analyses

Mean values (standard deviation) and count (frequencies) were used to describe continuous and categorical data, respectively. The Kruskal-Wallis test and one-way analysis of variance (ANOVA) were performed to assess the difference among subgroups with abnormal distribution and normal distribution, respectively. Categorical data were assessed with the Chi-square test and Cramer's V coefficient. Spearman's rank correlation was used to test the relationship between different variables when at least one of them was a rank variable. After univariate analysis, multivariate logistic regression was used to assess the significance of factors in MTM. A two-sided *P* < 0.05 was considered statistically significant. All statistical analyses were performed using commercial software (SPSS version 24.0; SPSS, Inc., Chicago, IL, United States).

## Results

### Demographics and Ocular Characteristics of Patients

From October 2018 to October 2020, a total of 723 highly myopic patients visited the ophthalmology clinic. Among them, 128 patients with 206 eyes had defined MTM, which met the criteria, and were further enrolled in the study with detailed analyses. Clinical characteristics of the eyes are summarized in Table 1. The mean age was 54.02 ± 11.5 years with a range of 17–79 years. Among the patients, 15 patients (11.72%) were younger than 40 years old. The proportion of female patients was higher than that of male patients (73.4 vs. 25.7%; Table 1).

**Table 1:** Demographic and ocular characteristics of patients with myopic tractional maculopathy.

**Variables**	**Patients**
Demographic characteristics
Age (y), mean ± SD	54.02 ± 11.5
Sex, *n* (%)
Male	53 (25.7%)
Female	153 (74.3%)
Ocular characteristics
BCVA in logMAR unit (Snellen), mean ± SD	0.69 ± 0.54 (20/29)
Axial length (mm), mean ± SD	29.86 ± 2.05
Intraocular pressure (mmHg), mean ± SD	15.32 ± 3.59
Myopic atrophy maculopathy, *n* (%)
Tessellated fundus (grade A1)	61 (29.6%)
Diffuse chorioretinal atrophy (grade A2)	88 (42.7%)
Patchy chorioretinal atrophy (grade A3)	40 (19.4%)
Macular atrophy (grade A4)	17 (8.3%)
Parapapillary atrophy
Gamma zone	206 (100%)
Delta zone	170 (82.52%)
Inner retinoschisis, *n* (%)	132 (64.1%)
Middle retinoschisis, *n* (%)	83 (40.3%)
Outer retinoschisis, *n* (%)	180 (87.4%)
Number of retinoschisis layers
One-layer retinoschisis	68 (33.0%)
Two-layer retinoschisis	72 (35.0%)
Three-layer retinoschisis	66 (32.0%)
Grade of outer retinoschisis
S0; no outer retinoschisis	21 (10.2%)
S1; extra foveal	31 (15.0%)
S2; foveal	24 (11.7%)
S3; foveal and extra foveal	33 (16.0%)
S4; entire macular	97 (47.1%)

*SD, standard deviation; BCVA, best corrected visual acuity*.

The results showed that as AXL rose, the grades of MAM increased (*r* = 0.446, *P* < 0.001). There was no association in AXL with the grades of outer retinoschisis and the number of retinoschisis layers (*r* = −0.048, *P* = 0.507; *r* = 0.019, *P* = 0.798). Besides, no significant difference in AXL between different grades of outer retinoschisis or different numbers of retinoschisis layers was detected (Chi-square value = 6.587, *P* = 0.159; *F* = 0.103, *P* = 0.902).

### The Association in MTM With Atrophic Changes

**Figure 1:**
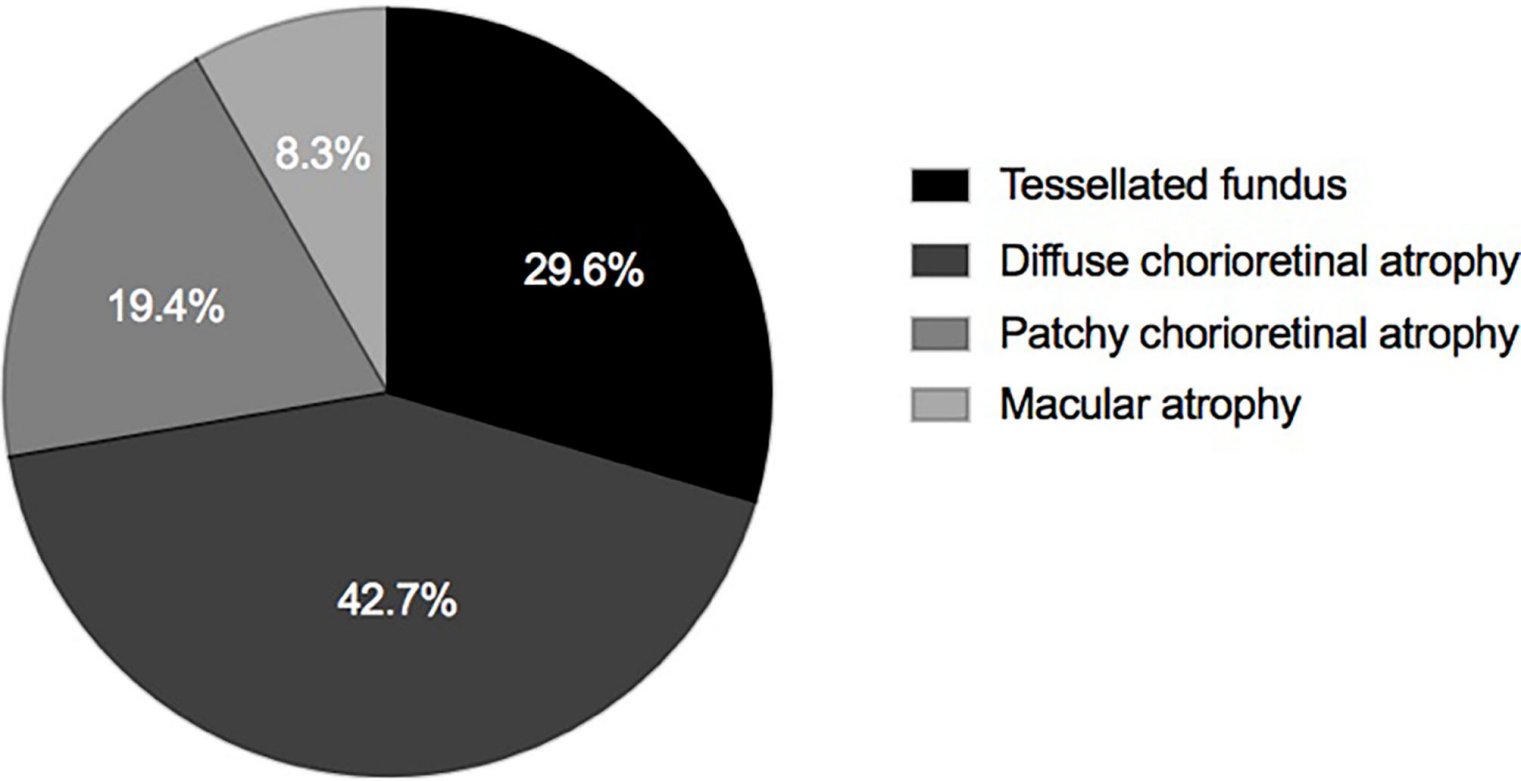
Distributions of myopic atrophy maculopathy in eyes with definite myopic tractional maculopathy.

**Figure 2:**
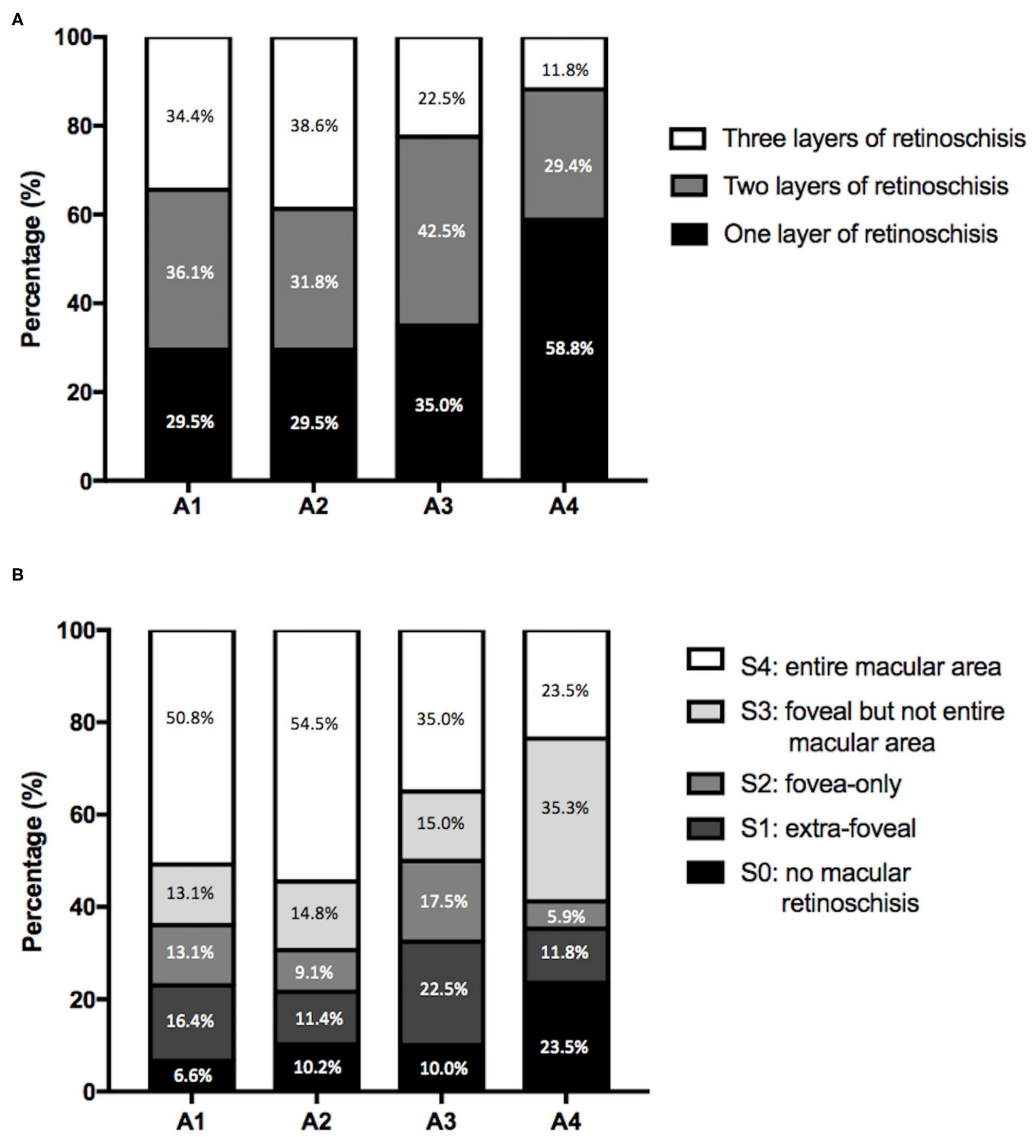
Features of myopic retinoschisis in different myopic atrophy maculopathy (MAM) grades. **(A)** Distributions of numbers of retinoschisis layers in different MAM grades. **(B)** Distributions of the range and location of outer retinoschisis in different MAM grades. A1, tessellated fundus; A2, diffuse chorioretinal atrophy; A3, patchy chorioretinal atrophy; A4, macular atrophy.

Of all the eyes, 61 (29.6%), 88 (42.7%), 40 (19.4%), and 17 (8.3%) had MAM grades of A1, A2, A3, and A4 (Figure 1 and Table 1). No eye belonged to A0. For number of retinoschisis layers, the incidence of three-layer retinoschisis was close in the MAM grades A1 (34.4%) and A2 (38.6%), peaked at A2, and then decreased. With the increasing MAM grades, the rate trend of entire macular retinoschisis was similar to that of three-layer retinoschisis, which was highest at A2 (54.5%), followed by A1 (50.8%). At A2, the incidence gradually declined (Figure 2 and Table 2). Both the number of retinoschisis layers and grades of outer retinoschisis presented a weakly negative association with the severity of MAM (*r* = −0.138, *P* = 0.048; *r* = −0.139, *P* = 0.047; Figure 2).

**Table 2:** Characteristics of myopic tractional maculopathy in different grades of myopic atrophy maculopathy.

	**Grades of myopic atrophy maculopathy**				***P***
	**A1, *n* (%)**	**A2, *n* (%)**	**A3, *n* (%)**	**A4, *n* (%)**	
**Number of retinoschisis layers**
One-layer retinoschisis	18 (29.5%)	26 (29.5%)	14 (35%)	10 (58.8%)	0.137
Two-layer retinoschisis	22 (36.1%)	28 (31.8%)	17 (42.5%)	5 (29.4%)	
Three-layer retinoschisis	21 (34.4%)	34 (38.6%)	9 (22.5%)	2 (11.8%)	
**Grade of outer retinoschisis**
S0; no outer retinoschisis	4 (6.6%)	9 (10.2%)	4 (10.0%)	4 (23.5%)	0.189
S1; extra foveal	10 (16.4%)	10 (11.4%)	9 (22.5%)	2 (11.8%)	
S2; foveal	8 (13.1%)	8 (9.1%)	7 (17.5%)	1 (5.9%)	
S3; foveal and extra foveal	8 (13.1%)	13 (14.8%)	6 (15.0%)	6 (35.3%)	
S4; entire macular	31 (50.8%)	48 (54.5%)	14 (35.0%)	4 (23.5%)	
**Complications of myopic retinoschisis**
Retinal detachments	15 (24.6%)	34 (38.6%)	10 (25.0%)	3 (17.6%)	0.128
Inner lamellar macular holes	12 (19.7%)	17 (19.3%)	6 (15.0%)	1 (5.9%)	0.457
Outer lamellar macular holes	8 (13.1%)	22 (25.0%)	4 (10.0%)	0 (0.0%)	0.007
Full thickness macular holes	2 (3.3%)	1 (1.1%)	5 (12.5%)	3 (17.6%)	0.010
**Paravascular abnormalities**
Paravascular microfolds	23 (37.7%)	47 (53.4%)	28 (70.0%)	8 (47.1%)	0.016
Paravascular retinal cysts	34 (55.7%)	66 (75.0%)	29 (72.5%)	9 (52.9%)	0.044
Paravascular lamellar holes	7 (11.5%)	15 (17.0%)	7 (17.5%)	4 (23.5%)	0.619

*P, Chi-square test*.

*A1, tessellated fundus; A2, diffuse chorioretinal atrophy; A3, patchy chorioretinal atrophy; A4, macular atrophy (8)*.

Here, we took gamma zones and delta zones to evaluate parapapillary atrophic changes in MTM. All the eyes had gamma zones, and 170 (82.5%) eyes showed delta zones simultaneously. The incidence of delta zones in the eyes with MTM was 73.8, 84.1, 90.0, and 88.2%, from the MAM grade A1–A4, respectively. There was no significant difference in the incidence of delta zones between different grades of outer retinoschisis or different numbers of retinoschisis layers (*P* = 0.764; *P* = 0.619).

### The Association in Complications of MR With MAM

**Figure 3:**
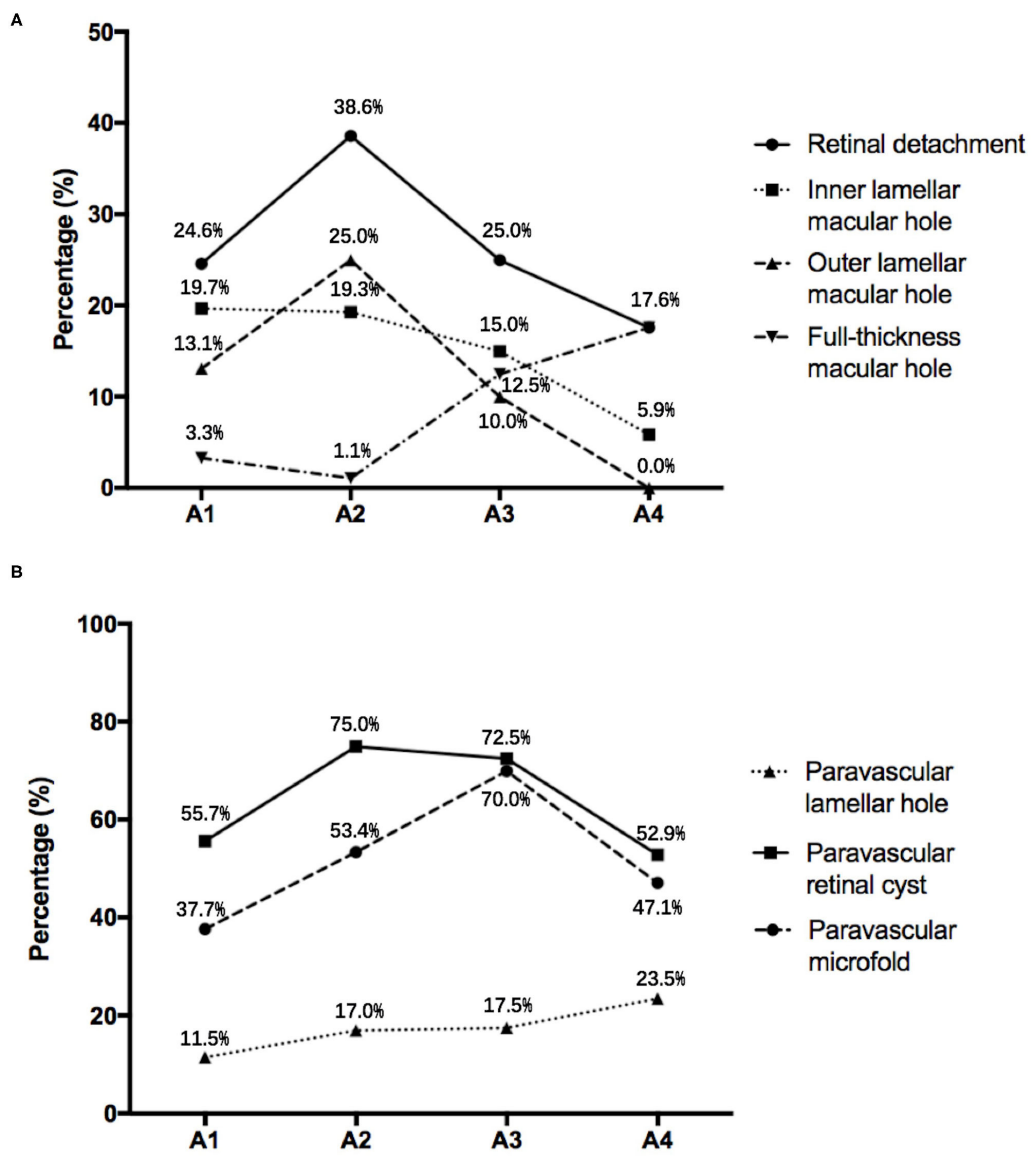
**(A)** The incidence of complications of myopic retinoschisis in different myopic atrophy maculopathy (MAM) grades. **(B)** The incidence of paravascular abnormalities in different stages of MAM. A1, tessellated fundus; A2, diffuse chorioretinal atrophy; A3, patchy chorioretinal atrophy; A4, macular atrophy.

In the study, the prevalence of ILMH, OLMH, and FTMH in the highly myopic eyes was 17.5, 16.5, and 5.3%, respectively (Table 2). The prevalence of ILMH went down as the severity of MAM increased. The incidence of OLMH and FTMH showed an opposite trend with the grade of MAM. The OLMH rate reached its peak at A2 and then decreased gradually. On the contrary, the incidence of FTMH reached its minimum at A2 and then increased (Figure 3A). The result showed that the OLMH rate weakly correlated with the grade of MAM (Cramer's V coefficient = 0.217; *P* = 0.007). The prevalence of OLMH in MR eyes with A1 and A2 was significantly higher than that in MR eyes with A3 and A4 (20.1 vs. 7.0%, *P* = 0.023). Besides, FTMH also presented a weak correlation with MAM (Cramer's V coefficient = 0.249; *P* = 0.010). The MR eyes with MAM grades A3 and A4 had a higher rate of FTMH than those with MAM grades A1 and A2 (14.0 vs. 2.0%, *P* = 0.002).

Given that myopic maculopathy might be affected by age, gender, and AXL, we conducted univariate analyses to test the association of these factors with the occurrence of OLMH and FTMH (Table 3). According to the result, we selected age (*P* < 0.10) and the severity of MAM as covariates to perform multivariable regression analysis for OLMH and FTMH. Compared with the MR eyes with A3 and A4, the results showed that A1 and A2 were more likely to have OLMH (*P* = 0.028; OR, 3.423; 95% CI, 1.144–10.236, Table 4). On the contrary, compared with A1 and A2, A3, and A4 were inclined to have FTMH (*P* = 0.004; OR, 7.752; 95% CI, 1.951–30.803; Table 4).

**Table 3:** Univariate analysis of systemic and ocular characteristics for outer lamellar macular holes and full-thickness macular holes.

	**Odds ratio (95% confidence interval)**	***P***
**Outer lamellar macular hole**
Age (y)	1.006 (0.973–1.040)	0.717
Gender (female)	1.152 (0.486–2.728)	0.748
Axial length (mm)	1.029 (0.859–1.232)	0.757
**Full-thickness macular hole**
Age (y)	1.053 (0.992–1.117)	0.087
Gender (female)	3.636 (0.454–29.107)	0.224
Axial length (mm)	1.210 (0.906–1.616)	0.197

**Table 4:** Multivariable analysis for outer lamellar macular holes and full-thickness macular holes.

	**Odds ratio (95% confidence interval)**	***P***
**Model for outer lamellar macular holes**
Age (y)	1.010 (0.977–1.043)	0.557
Myopic atrophy maculopathy (A1 + A2)	3.423 (1.144–10.236)	0.028
**Model for full-thickness macular hole**
Age (y)	1.057 (0.988–1.131)	0.109
Myopic atrophy maculopathy (A3 + A4)	7.752 (1.951–30.803)	0.004

*A1, tessellated fundus; A2, diffuse chorioretinal atrophy; A3, patchy chorioretinal atrophy; A4, macular atrophy (8)*.

Sixty-two of the eyes (30.1%) presented with RD. As the severity of MAM increased, the trend of RD rate was similar to that of OLMH. Overall, 79% of RD belonged to MAM grades A1 and A2.

### The Association in PVAs With MAM

For different kinds of PVAs, paravascular retinal cysts had the highest incidence (67.0%), followed by paravascular microfolds (51.5%) and then paravascular lamellar holes (16.0%). The incidence of retinal cysts and microfolds had a weak correlation with MAM (Cramer's V = 0.198, *P* = 0.044; Cramer's V = 0.225, *P* = 0.016; Table 2). The eyes with MAM grades A2 and A3 were more likely to have retinal cysts and microfolds than those with MAM grades A1 and A4 (74.2 vs. 55.1%, *P* = 0.005; 58.6 vs. 39.7%, *P* = 0.009). However, the rate trends of these two kinds of PVAs were still slightly different, with the increase of MAM grades. The incidence of retinal cysts was highest in A2 and then decreased with the severity of MAM rising. While the rate of paravascular microfolds reached the highest in A3 and then went down. The prevalence of paravascular lamellar holes showed an upward trend from A1 to A4 (Figure 3B). In addition, the varieties of PVAs had a positive correlation with the grade of MAM (*r* = 0.165, *P* = 0.018) and AXL (*r* = 0.179, *P* = 0.013).

## Discussion

As far as we know, this is the first time the atrophic features in eyes with definite MTM have been elaborately described. Diffuse chorioretinal atrophy occupied the most significant MAM grades in eyes with MTM, followed by tessellated fundus. These two kinds of MAM grades accounted for the vast majority in eyes with MTM (72.3%). More extensive and multi-layered forms of retinoschisis might be likely to occur in less severe MAM. More than 80% of eyes with MTM had both gamma zones and delta zones. In addition, we found that atrophic changes might be involved in the development of MH. The eyes with tessellated fundus and diffuse chorioretinal atrophy were more likely to have OLMH, and those with well-defined atrophy were more likely to present with FTMH. With MAM aggravation and AXL growth, the types of PVAs increased. To more comprehensively assess the relationship between tractional and atrophic features, we chose multiple perspectives to reflect the characteristics of MTM, like the range and location of outer retinoschisis, different layers of retinoschisis, various complications, and PVAs rather than the tractional component in the ATN classification (8).

Previous studies have shown that being female was a risk factor for myopic maculopathy (10, 17). Here, the percentage of female patients was higher than male patients. To reflect the characteristics of MTM as much as possible, we did not limit the age of the subjects to 50 years or older. While the mean age in our study was also nearly to that of previous studies (4, 13). Moreover, around 10% of patients with MTM were younger than 40 years old.

From the integrity of ocular changes in pathologic myopia, atrophic lesions involve both macular and parapapillary zones. In the study, we briefly analyzed parapapillary atrophy in MTM and found that more than 80% of eyes with MTM had both gamma zones and delta zones. Moreover, even in MR eyes with tessellated fundus, 73.8% of them had both kinds of parapapillary atrophies, representing severe parapapillary atrophy. Besides, a delta zone is from the elongated and thinned peripapillary scleral flange, which implies significant remodeling of the ocular shape (11). Therefore, the high prevalence of delta zones may also suggest the effect of the sclera shape in MTM development.

In 2003, Baba et al. assessed 134 eyes with high myopia and found out of 7 eyes with foveal detachment or retinoschisis all had well-defined chorioretinal atrophy. So they believed retinoschisis always developed in eyes with severe MAM (9). In the study by Chen et al. the severity of MTM was not in keeping with MAM based on the ATN classification (10). Later, Takahashi et al. further demonstrated that a shorter AXL might be a risk factor for MR (12). Here, we found that above 70% of eyes with MTM had diffuse chorioretinal atrophy and a tessellated fundus. Besides, the severity of MR presented a weak negative correlation with grades of MAM, which were in indirect agreement with previous studies (12).

Further deliberating the distributions of layers and grades of MR and the complication rates in different stages of MAM, we found that all the incidences of three layers of retinoschisis, entire macular retinoschisis, RD, and OLMH reached the highest in the MAM grade A2 (Figures 2, 3). Besides, the eyes with a tessellated fundus and diffuse chorioretinal atrophy were more likely to have OLMH, and those with more advanced stages of MAM were more likely to present with FTMH. Some studies laid out detailed descriptions about the natural course from retinoschisis to the complication stage and referred to the significance of vitreous traction and premacular structure (18–20). Parolini et al. believed the natural evolution of MR depended on two forces, the perpendicular force to the retina which induced retinoschisis and FD, and the tangential force to the retina which induced MH (21).

**Figure 4:**
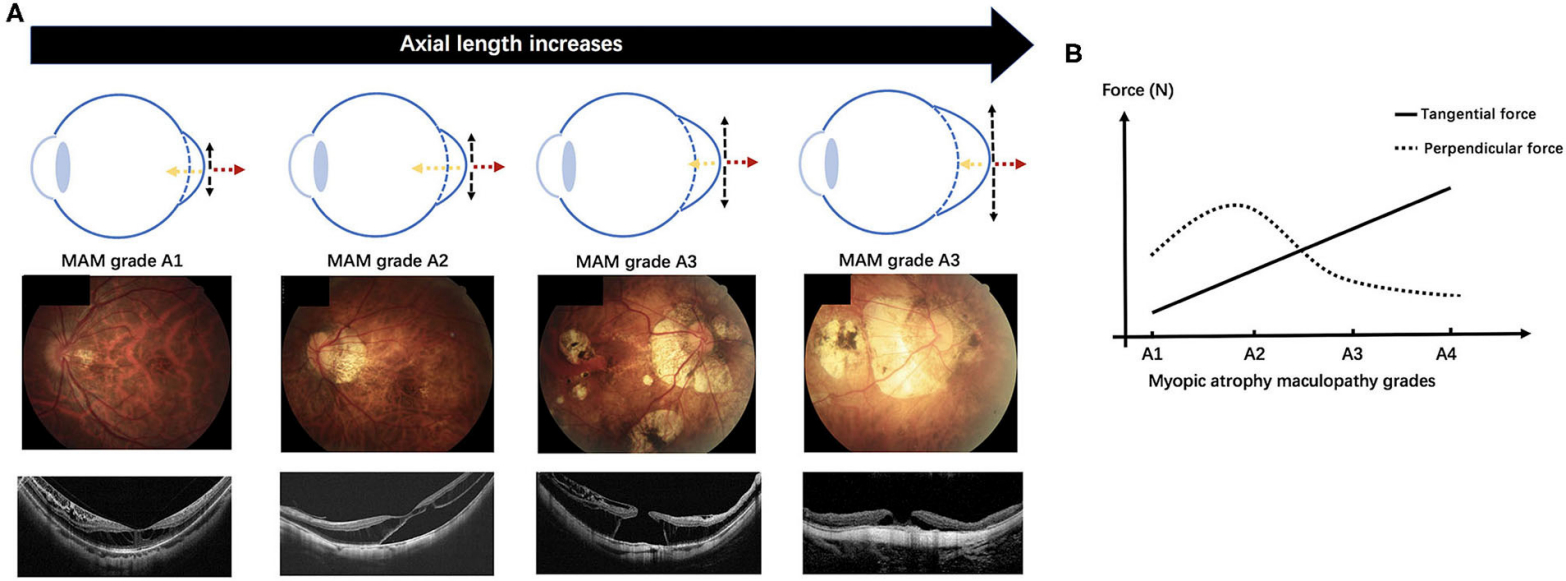
We proposed a hypothesis on the mechanics-related changes of pathologic myopia based on the study. In the early stage of pathologic myopia, the macular is mainly affected by the perpendicular force, which gradually increases under the inward pulling force of the vitreous and the backward pulling force of posterior sclera extension, and reaches the highest at the myopic atrophy maculopathy (MAM) grade A2. After A2, the vitreous might be liquefied and reduce the inward force. The summation of perpendicular force falls and the tangential force gradually increases becoming the dominant factor causing retinopathy. **(A)** A schematic diagram demonstrates the change of the perpendicular force and the tangential force acting on the macular with the axial length growth (the top row). Fundus images with increasing MAM grades (the middle row) and optical coherence tomography images which show the corresponding myopic tractional maculopathy (the bottom row). **(B)** The variation trend of the perpendicular force and the tangential force with MAM grades increasing in the hypothesis. Myopic atrophy maculopathy, MAM; A1, tessellated fundus; A2, diffuse chorioretinal atrophy; A3, patchy chorioretinal atrophy; A4, macular atrophy. Black dotted arrow, the tangential force; yellow dotted arrow, the inward pulling force of the vitreous; red dotted arrow, the backward pulling force of the posterior sclera extension.

**Figure 5:**
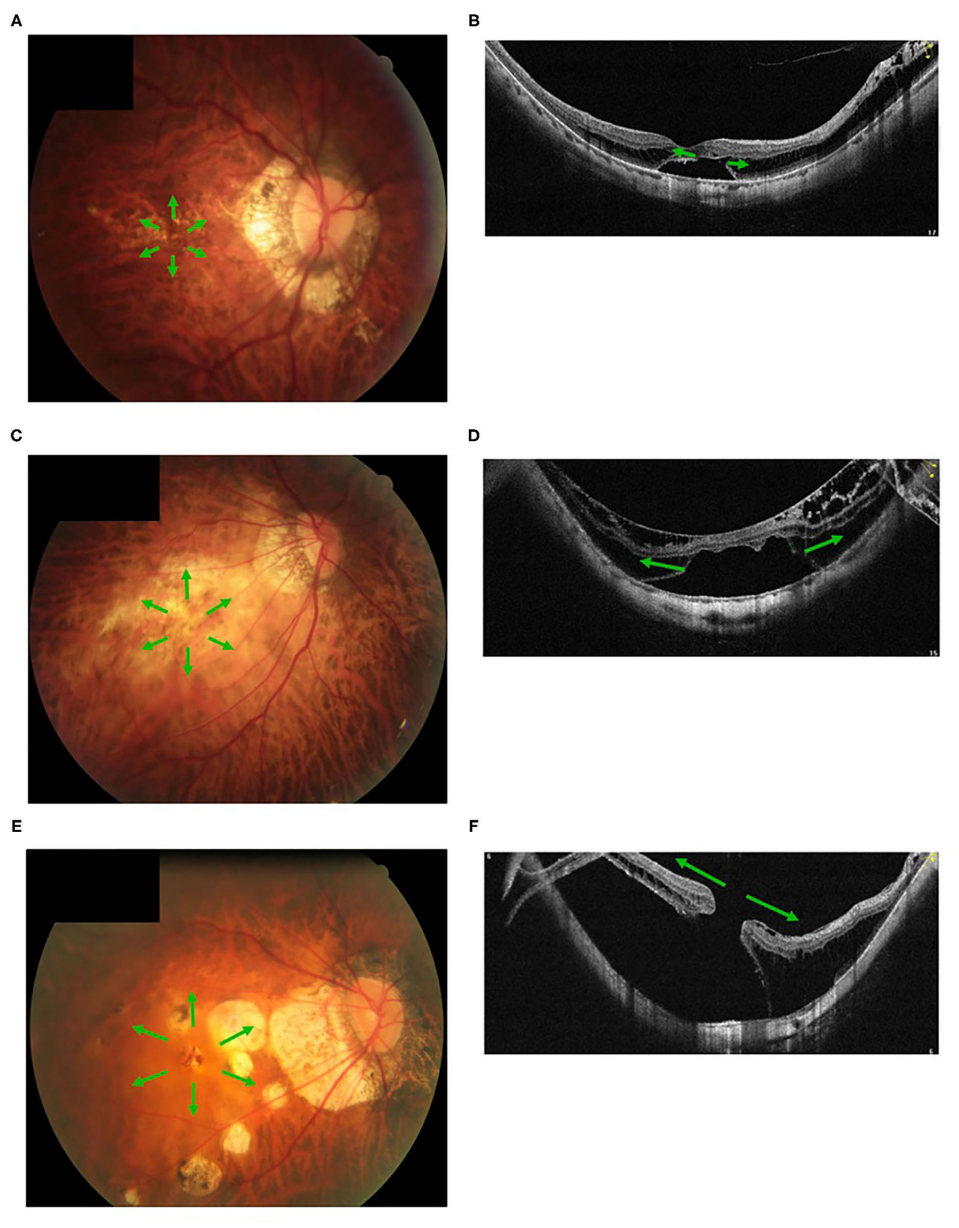
Fundus images with different myopic atrophy maculopathy grades and corresponding myopic tractional maculopathy in optical coherence tomography. A patient with mild diffuse chorioretinal atrophy **(A)** presented with a small outer lamellar macular hole and foveal detachment **(B)**. A patient with more serious diffuse chorioretinal atrophy **(C)** presented with a large outer lamellar macular hole and foveal detachment **(D)**. A patient with patchy chorioretinal atrophy **(E)** presented with a full-thickness macular hole and retinal detachment **(F)**. Green arrow, the tangential traction on the macular might facilitate a lamellar macular hole into a full-thickness macular hole.

Based on our results and the view proposed by Parolini et al. we guess the atrophic process might involve developing complications and propose a hypothesis on the mechanics-related progression of MAM and MTM. A schematic diagram is shown in Figure 4. In the progression of pathological myopia, the force on the macula can be mainly divided into a perpendicular force and a tangential force. The tangential force, which promotes atrophic changes, is mainly induced by AXL elongation. With the growth of AXL, the tangential force increases, and atrophy maculopathy aggravates. The perpendicular force, which induces traction maculopathy, is mainly composed of the inward pulling force of the vitreous and the backward pulling force generated during posterior sclera extension. In the early stage of pathologic myopia, the macular is mainly affected by the perpendicular force, which gradually increases under two opposite tractions and reaches the highest at A2. After A2, the vitreous might be liquefied and reduce the force that pulls the macular inward. Thus, the summation of perpendicular force falls. In the meantime, the tangential force gradually increasing becomes the dominant factor causing retinopathy. As a complicated stage, MAM grade A2 endures the incremental perpendicular force and tangential force simultaneously. That can explain why severe MR, RD, and OLMH have the highest incidences in A2. After that, the augmented tangential force further causes lamellar MH to develop into FTMH (Figure 5).

Recent studies have proved that PVAs represented traction on the retina and were a risk factor for retinoschisis (12, 15, 22, 23). However, on the sequence of different kinds of PVAs, people have different views. Shimada et al. believed paravascular cysts were the first (15). But Kamal-Salah et al. found more paravascular microfolds than paravascular cysts and proposed that paravascular microfolds were earlier (22). From the view of MAM progression, our study showed that more paravascular retinal cysts became apparent in an early stage than paravascular microfolds. Paravascular microfolds could represent the inward force and form paravascular cysts. However, retinoschisis itself can also lead to cysts near vessels. Therefore, we speculated that paravascular retinal cysts appear before microfolds. Paravascular lamellar holes might come from the breakage of paravascular cysts (15, 24). Li et al. believed that paravascular lamellar holes were the advanced stage of PVAs because all the eyes with paravascular lamellar holes had paravascular microfolds and cysts simultaneously (23). In our study, the incidence of paravascular lamellar holes was the least and presented an upward trend with MAM aggravation. Therefore, we also believe the paravascular lamellar hole was the last to appear, keeping with the previous study (23).

Limitations in the study need to be noted. As a cross-sectional study, it is hard to directly reflect the relationship between the progression of the two types of myopic maculopathy. The order in which different kinds of PVAs occur was inferred from the current analysis and needs further verification. To obtain more analyzable data about MTM, we conducted the study based on hospital recruitment. Therefore, some patients with good vision and early stages of MR might be missing. A large, longitudinal population study can provide more sound evidence for the correlation between MAM on MTM. Based on the study, we made a preliminary assumption on the mechanics-related changes of pathologic myopia and the interaction between MAM and MTM. The hypothesis still has many deficiencies and needs to be proved from many aspects.

In conclusion, this is the first study to demonstrate the features of atrophic changes in eyes with definite MTM. Combining MAM and MTM for a comprehensive analysis leads to a deeper understanding of the characteristics of MTM as well as the mechanism of pathologic myopia. For eyes with MTM, diffuse chorioretinal atrophy was the dominant MAM grade, followed by a tessellated fundus. Over 70% of the eyes presented with these two grades of MAM. In clinical management, even if a highly myopic patient presents with an early stage of MAM, an OCT scan is necessary for excluding MTM, especially when delta zones co-exist. Given that the three layers of retinoschisis, entire macular retinoschisis, RD, and OLMH have the highest incidence in MAM grade A2, diffuse chorioretinal atrophy might be a complicated stage for MTM. Besides, the progression of MAM might involve the development of MH. When MTM combines with well-defined atrophy, FTMH should be noted. According to the study, we proposed a preliminary hypothesis on the mechanics-related changes of pathologic myopia and the interaction between MAM and MTM. Here, we found weakly negative correlations in grades of MAM with the range and layers of MR. The relationship still needs to be demonstrated in the future.

## Data Availability Statement

The original contributions presented in the study are included in the article/supplementary material, further inquiries can be directed to the corresponding authors.

## Ethics Statement

The studies involving human participants were reviewed and approved by Ethics Committee of Beijing Tongren Hospital. Written informed consent from the participants' legal guardian/next of kin was not required to participate in this study in accordance with the national legislation and the institutional requirements.

## Author Contributions

JT, YQ, and NW: study concept and design and manuscript revision. JT, CL, and YQ: performed study. JT and YQ: drafted manuscript. JT and KC: statistical analysis. NW: administrative, technical, material support, or study supervision. All authors participated in and provided help for the study.

## Conflict of Interest

The authors declare that the research was conducted in the absence of any commercial or financial relationships that could be construed as a potential conflict of interest.

## Publisher's Note

All claims expressed in this article are solely those of the authors and do not necessarily represent those of their affiliated organizations, or those of the publisher, the editors and the reviewers. Any product that may be evaluated in this article, or claim that may be made by its manufacturer, is not guaranteed or endorsed by the publisher.
